# Adaptive specialization of a unique sponge body from the Cambrian Qingjiang biota

**DOI:** 10.1098/rspb.2022.0804

**Published:** 2022-06-29

**Authors:** Hao Yun, Cui Luo, Chao Chang, Luoyang Li, Joachim Reitner, Xingliang Zhang

**Affiliations:** ^1^ State Key Laboratory of Continental Dynamics and Shaanxi Key Laboratory of Early Life and Environments, Department of Geology, Northwest University, Xi'an 710069, People's Republic of China; ^2^ State Key Laboratory of Palaeobiology and Stratigraphy, Nanjing Institute of Geology and Palaeontology and Center for Excellence in Life and Paleoenvironment, Chinese Academy of Sciences, Nanjing 210008, People's Republic of China; ^3^ Key Laboratory of Submarine Geosciences and Prospecting Techniques, Ministry of Education, and College of Marine Geosciences, Ocean University of China, Qingdao 266100, People's Republic of China; ^4^ Department of Geobiology, Centre of Geosciences of the University of Göttingen, Goldschmidtstraße 3, Göttingen 37077, Germany

**Keywords:** Hexactinellida, adaptative evolution, black shale, Cambrian, Qingjiang biota

## Abstract

Sponge fossils from the Cambrian black shales have attracted attention from both palaeontologists and geochemists for many years in terms of their high diversity, beautiful preservation and perplexing adaptation to inhospitable living environments. However, the body shape of these sponges, which contributes to deciphering adaptive evolution, has not been scrutinized. New complete specimens of the hexactinellid sponge *Sanshapentella tentoriformis* sp. nov. from the Qingjiang biota (black shale of the Cambrian Stage 3 Shuijingtuo Formation, *ca* 518 Ma) allow recognition of a unique dendriform body characterized by a columnar trunk with multiple conical high peaks and distinctive quadripod-shaped dermal spicules that frame each high peak. The body shape of this new sponge along with other early Cambrian hexactinellids, is classified into three morpho-groups that reflect different levels of adaptivity to the environment. The cylindrical and ovoid bodies generally adapted to a large spectrum of environments; however, the dendriform body of *S. tentoriformis* was restricted to the relatively deep-water, oxygen-deficient environment. From a hindsight view, the unique body shape represents a consequence of adaptation that helps maintain an effective use of oxygen and a low energy cost in hypoxic conditions.

## 1. Introduction

Molecular clock analyses and lipid biomarker data indicate that sponges probably arose before 750 million years ago (Ma) [1–4], however, except for some problematic Ediacaran sponges such as *Thectardis* [5] and *Coronacollina* [6], the oldest conclusive sponge fossils were much younger in age, near the Precambrian–Cambrian transition (*ca* 540 Ma) [7–10]. Thereafter, the sponge group, along with other metazoans, diversified rapidly during the Cambrian Explosion [11]. The Cambrian sponges are represented by numerous exceptionally preserved body fossils and isolated spicules from all over the world [12–21]. They occurred in a wide range of environmental settings [22,23] while were particularly dominant in the early Cambrian (Terreneuvian to Epoch 2, *ca* 540–510 Ma) black shales in South China compared with other animal groups [12,14,24,25].

The black shales generally possess high total organic carbon contents (greater than 2% weight) and indicate a shelf margin to slope environment with dysoxic to anoxic (even euxinic) bottom waters [26–28]. Therefore, the environment dominated by black shales is inhospitable for most benthic animals [29]. Recent physiological experiments have suggested that modern sponges can survive in (but mostly not adapt to) low-oxygen conditions of 0.5% to 4% present atmospheric level [30,31] and anaerobic microbial symbionts could help some taxa cope with low oxygen conditions [32]. However, it is still perplexing how a series of prosperous sponge communities managed to develop in the oxygen-deficient Cambrian black shales. For many years, various hypotheses from environmental perspectives were proposed to interpret the phenomenon, such as obtaining a supply of nutrients and oxygen through hydrothermal venting, upwelling seawater, occasional currents and so on [25,33–35], whereas the adaptability and ecological strategies of the animals have rarely been considered.

There are at least 17 sponge genera that have been discovered in the Cambrian black shales (Niutitang, Shuijingtuo, Hetang and Huangboling formations) of South China [14,22,23,25]. Most of them were restored from well-preserved body fossils and integrated skeletal frameworks; however, some genera, such as *Sanshapentella* Mehl and Erdtmann, 1994 and probably *Solactiniella* Mehl and Reitner in Steiner *et al*., 1993, were described based only on incomplete fossils and isolated spicules [12,14,24,25,36,37]. Hence, more fossil materials and related studies are needed to decipher the morphology, taxonomy and phylogeny of these early sponge groups. In addition, almost all previous studies focused on the spicule structure and arrangement. The function of body shapes, however, has received scant attention.

In this study, complete body fossils of a new species of *Sanshapentella* (Hexactinellida, Porifera) from the Qingjiang biota (black shale of the Shuijingtuo Formation, *ca* 518 Ma [35]) are systematically studied and the redox condition of related palaeoenvironment is analysed by using geochemical proxies. Body shapes of early Cambrian sponges are scrutinized for the first time, to our knowledge, to investigate their adaptive evolution.

## 2. Material and methods

### (a) Fossil analysis

A total of 63 specimens of the new sponge species were collected from the Tianzhushan section of the Qingjiang biota (see the electronic supplementary material, text S1 and figure S1 for geological setting), 15 of them are preserved with relatively complete bodies. Fossils were photographed with a Canon EOS 5D Mark II camera under magnesium light. The obtained images were processed and preliminarily analysed with CorelDRAW X8 and Adobe Photoshop CS5 Extended. Secondary electron imaging and energy dispersive spectroscopic analyses with an FEI Quanta 450 scanning electron microscope and X-ray fluorescence (XRF) with a Bruker's M4 TORNADO spectrometer were performed to investigate the elemental composition of the fossils. Computed X-ray microtomography (micro-CT) under a Phoenix V Tome XM was used to reveal the internal structure of the specimens. The micro-CT data were processed by using VG Studio Max 3.2 for three-dimensional volume rendering.

### (b) Geochemical analysis

Rock powders for geochemical analysis were collected from the same bedding plane surrounding the studied sponge specimens (electronic supplementary material, table S1). Fe speciation was measured at the State Key Laboratory of Biogeology and Environmental Geology, China University of Geosciences (Wuhan), using the methods described in Poulton *et al*. [38]. The highly reactive Fe (Fe_HR_) includes pyrite (Fe_py_), carbonate-associated Fe (Fe_carb_), magnetite (Fe_mag_) and Ferric oxides (Fe_ox_). Fe_py_ was calculated based on the content of pyrite sulfur extracted as Ag_2_S following the Cr-reduction method [39], whereas Fe_carb_, Fe_mag_ and Fe_ox_ were measured through the sequential extraction procedure [38]. Concentrations of major and trace elements were analysed by inductively coupled plasma-mass spectrometry at the ALS Chemex (Guangzhou) Co., Ltd, following a four-acid digestion with HNO_3_, HClO_4_, HF and HCl. An internal standard solution of rhodium was added to monitor matrix effects and instrument drift. The relative analytical errors were less than 10% for both major and trace elements. Enrichment factors (EF) for trace elements (X) were calculated relative to continental crust (UCC; [40]) as: X_EF_ = (X/Al)_sample_/(X/Al)_UCC_.

## 3. Results

### (a) Systematic palaeontology

Phylum Porifera Grant, 1836

Class Hexactinellida Schmidt, 1870

Order and family uncertain

Genus ***Sanshapentella*** Mehl and Erdtmann, 1994

*Type species*. *Sanshapentella dapingi* Mehl and Erdtmann, 1994 from the Niutitang Formation (basal Stage 3 of Cambrian, *ca* 520 Ma) in Sancha, Hunan Province, China [37].

*Remarks*. Most articulated hexactinellid sponges from Cambrian biotas were settled under the morphological group Reticulosa Reid, 1958 that characterized by parallelly arranged stauractines, pentactines and hexactines [13,19,22,41–43]. Owing to the presence of definite hexactines and hexactine-derived spicules, *Sanshapentella* can be assigned to Hexactinellida [36,37]. However, the irregular rather than parallel arrangement of spicules and its possibility of possessing a thick body wall indicate that this genus does not conform to the skeletal construction of Reticulosa. 'Rosselimorpha' is another morphological group coined by Mehl [44] for ancient hexactinellids. The rosselimorph organization, exemplified by the Cambrian sponge *Solactiniella*, is characterized with a thick body wall, reduced forms of hexactines, and irregular distribution of these spicules [44]. *Sanshapentella* seems to be similar to *Solactiniella* in the irregular arrangement of spicules, while the dominance of triaxons and stauractines and a body confined by specific quadripod-shaped dermal pentactines differ it from the latter. By contrast, the spicule organization of *Sanshapentella* is most similar to the globular sponge fossils reported from phosphorous nodules of the basal Niutitang Formation in Sancha, Hunan Province [16], since they both possess irregularly arranged parenchymal (substrate) triaxons and dermal pentactines.

***Sanshapentella tentoriformis* sp. nov.**

(Figures 1 and 2)

**Figure 1.**
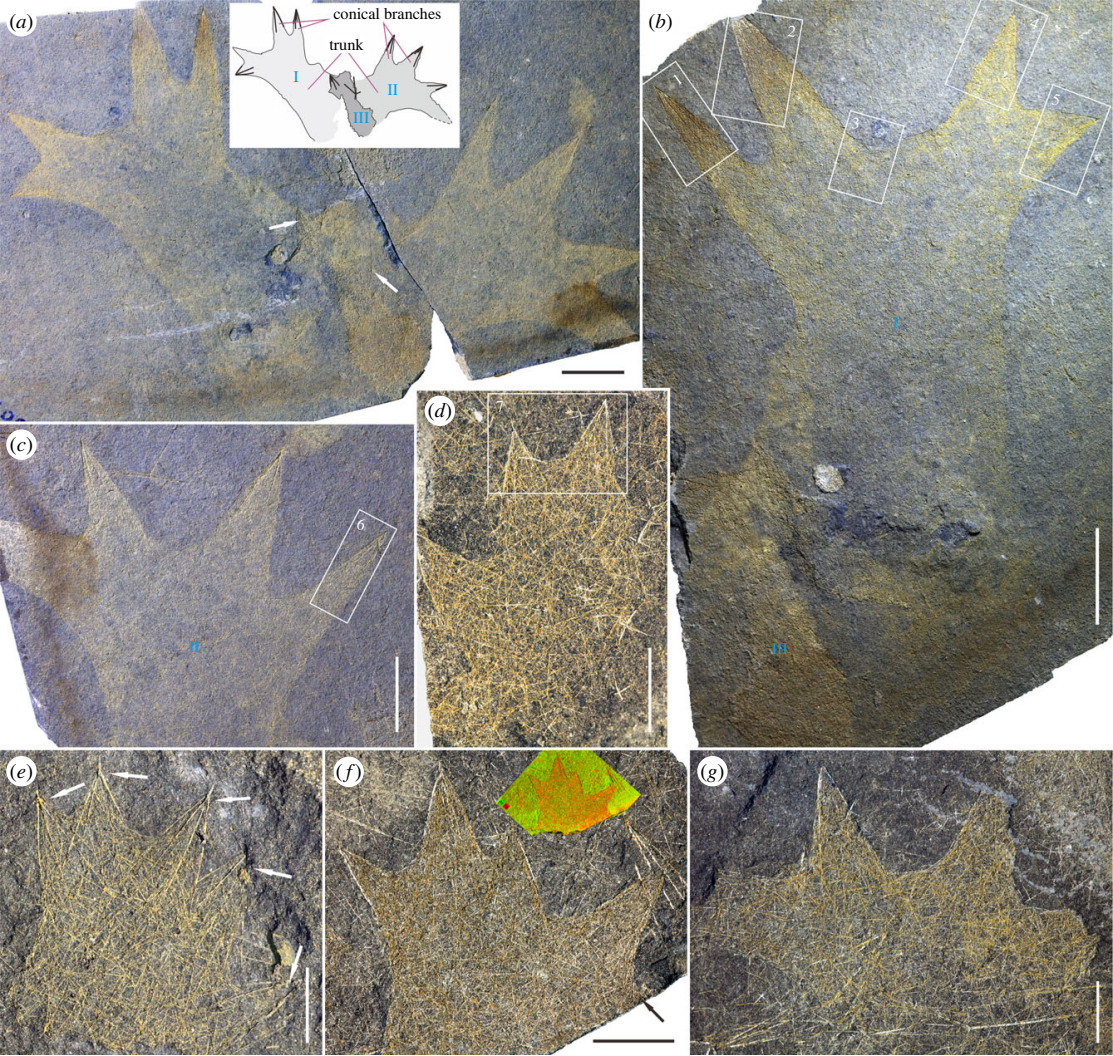
Complete bodies of *Sanshapentella tentoriformis* sp. nov. from the Qingjiang biota. (*a*) A rock slab revealing three partly superimposed specimens with distinctive dendriform bosies; white arrows indicating large dermal pentactines of the twisted specimen in the middle region. (*b*) Holotype (LELE-TZ-001) of *S. tentoriformis* (specimen I) and an incomplete, huddled specimen (III) at the lower left. (*c*) LELE-TZ-002 (specimen II). Specimens showing in (*b* and *c*) are counterparts of (*a*). (*d–g*) Representatives of relatively complete bodies with a smaller size but prominent conical branches; (*d*) LELE-TZ-037; (*e*) LELE-TZ-066, white arrows indicating five large dermal pentactines; (*f*) LELE-TZ-096, black arrow indicating the possible fifth branch; XRF image at the upper right showing concentration of Fe (red) in the fossil and Al (green; revealing aluminosilicates) in the background rock; and (*g*) LELE-TZ-070. Scale bars represent: 10 mm (*a–c*), 5 mm (*d*,*f*,*g*), 2 mm (*e*). (Online version in colour.)

**Figure 2.**
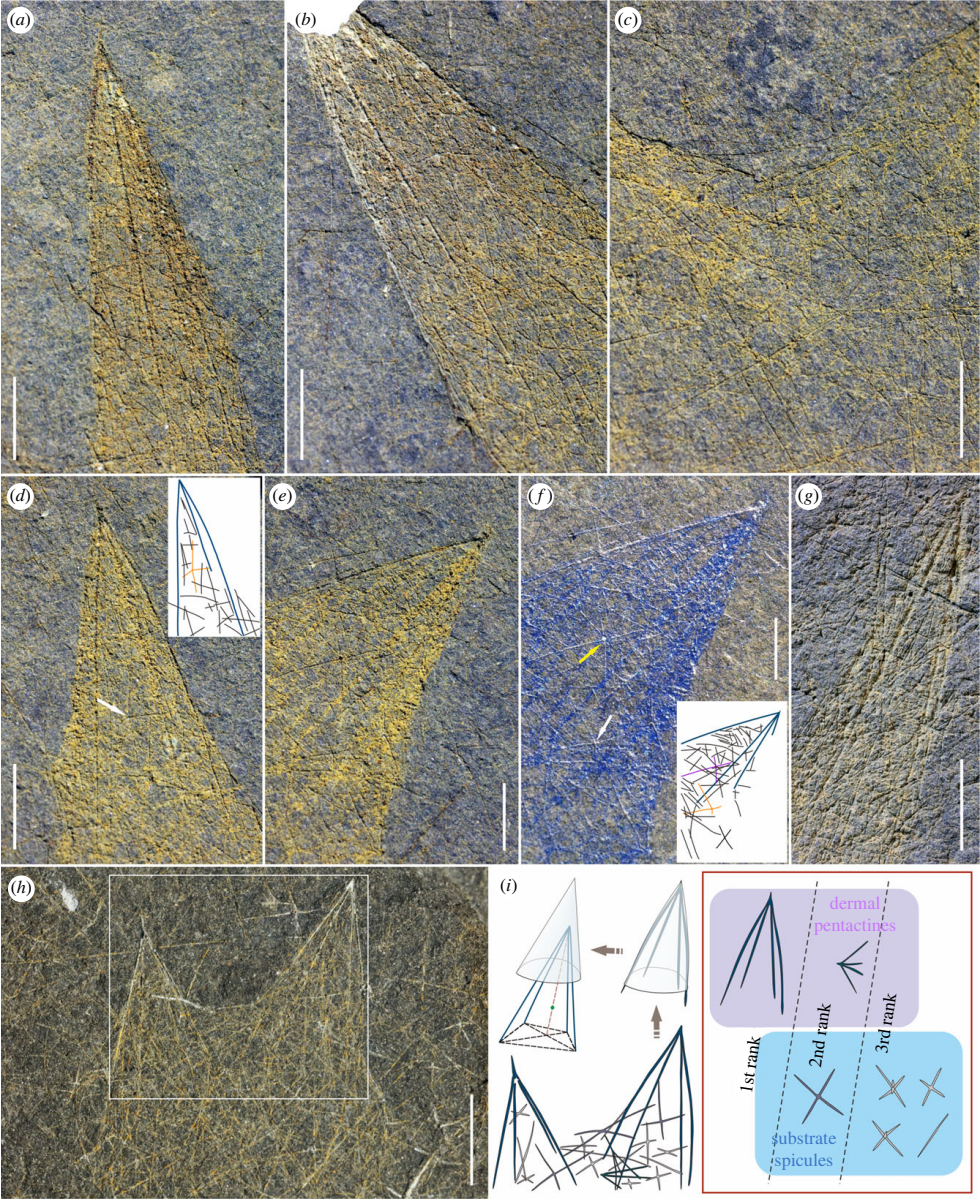
Spicules of *Sanshapentella tentoriformis* sp. nov. from the Qingjiang biota. (*a–e*) Details of the holotype showing prominent large dermal pentactines framing the conical branches and substrate triaxons filling the body surface; the positions are indicated by white frames 1–5, respectively, in figure 1*b*. (*f*) Dyeing image of (*e*) revealing hexactines and pentactines. (*g*) Detail of a conical ray of LELE-TZ-002; the position is indicated by the white frame 6 in figure 1*c*. White arrows and orange lines in the sketches of (*d* and *f*) indicate small dermal pentactines scattered on the body surface; yellow arrows and purple lines in the sketch of (*f*) indicate substrate hexactines. (*h*) Detail of LELE-TZ-037 showing clear spicule structures and arrangement patterns; the position is indicated by the white frame 7 in figure 1*d*. (*i*) Rough sketch of spicules in the white frame in (*h*) and interpretation and classification of spicules; the large dermal pentactines of the conical branches form a pyramid-like skeleton that have a relatively low centre of gravity (green point) and composed of four triangles. Scale bars represent: 2 mm. (Online version in colour.)

2005 Undetermined form 2; (Xiao *et al*. [24, p.109] fig. 10A–B).

*Etymology*. From the Latin *tentorium* (tent-shaped), to describe the shape of the distinctive conical branches.

*Holotype.* LELE-TZ-001 from the Tianzhushan section of the Qingjiang biota in Changyang, Hubei Province, People's Republic of China.

*Occurrence*. The lower Shuijingtuo Formation (Qingjiang biota, Cambrian Stage 3) in Hubei Province and the basal Hetang Formation (Cambrian Stage 2–3) in Hunan Province, People's Republic of China.

*Diagnosis*. Sponge body is composed of a stout trunk (usually 10–35 mm in thickness) and four or five conical branches at the top. The spicules include characteristic quadripod-shaped dermal pentactines and a dense mesh of hexactines, stauractines, diactines and other hexactine-derived spicules. Spicules exhibit three ranks of sizes. The largest (5–19 mm) are quadripod-shaped pentactines that form the frame of the conical branches. The second rank (2.5–7.5 mm) are pentactines and stauractines scattering on the body surface. The smallest (1–3 mm) are densely distributed triaxons, stauractines and diactines.

*Description*. The holotype, and a paratype (LELE-TZ-002) preserved adjacent to and partly superimposed with the holotype, both have a distinctive dendriform body shape and similar sizes (figure 1*a–c*). A third specimen, twisted and a smaller size, is positioned between the two specimens (figure 1*a*). The complete body of the sponge can be subdivided into two parts: a stout trunk and a crown composed of four tent-like high peaks (named as conical branches). The total height of the holotype is 70 mm, in which the trunk is 42.5 mm high. The trunk is 28 mm in width while the crown is 50 mm wide at the widest region. The four conical branches (heights of the cones are 9, 12.5, 20 and 22 mm, respectively) of the crown can be grouped into two pairs (left pair and right pair), and these two pairs are approximately bilaterally symmetrical to each other. Including the holotype and the paratype LELE-TZ-002, there are 15 specimens preserved with relatively complete bodies or skeletal frameworks (figure 1*d–g*). Most specimens (12 of the 15 complete bodies) have four conical branches, however, a few of the bodies, such as LELE-TZ-066, LELE-TZ-096 and LELE-TZ-098 possess five (figure 1*e,f*; electronic supplementary material, figure S2*c*).

Two morphological series of spicules are recognized, including dermal pentactines that partly protrude out from the body and abundant substrate spicules that form the dense skeletal mesh (figures 1 and 2). The dermal pentactines are quadripod-shaped, composed of a short ray pointing outwards and four long paratangential rays that bend towards the body. The substrate spicules, including diactines, stauractines, hexactines and hexactine-derived pentactines, are densely distributed and interlaced in both the trunk and the conical branches.

The spicules can be classified into three ranks by size (figure 2*i*; electronic supplementary material, table S2). The first rank, ranging from 5 to 19 mm except in two small (juvenile) specimens, are the particularly large dermal pentactines; each of them serves as an overall framework of a conical branch (figure 2*a*,*b*,*d–h*). Paratangential rays of the large pentactines in the holotype, 8–13.5 mm in length and 0.1 mm in diameter, are arranged along the surface of each branch, reminiscent of the frame of a pagoda tent. The outward-pointing short rays are degenerated and no more than 0.5 mm long, forming the apex of the branch. The second rank, mostly 2.5–7.5 mm in size, is represented by a series of smaller dermal pentactines and substrate stauractines (and maybe some hexactines) that are scattered on the body surface. The paratangential rays of the small dermal pentactines in the holotype are 2.5–3.5 mm in length (figure 2*d*,*f*, indicated by white arrows); and the tangential rays of the stauractines are 3–5.5 mm in length (figure 2*c*,*f*). The third rank, 1–3 mm in size, includes most substrate triaxons, stauractines and diactines in the skeletal mesh that generally have a small size. The ray length of these small spicules in the holotype mostly ranges from 1.5 to 3 mm (figure 2*a–g*). The arrangement of the substrate spicules is relatively irregular, though the second-rank stauractines seem to have rays usually obliquely crossing the main axis of the sponge body (figure 2*c*,*e*,*h*). Additionally, the conical branches framed by large dermal pentactines, as well as small triaxons, are not only observed on the bedding surface of sediments but also revealed inside the rock samples and some of the spicules are preserved three-dimensionally (electronic supplementary material, figure S3 and video S1).

### (b) Redox condition of the living environment

**Figure 3.**
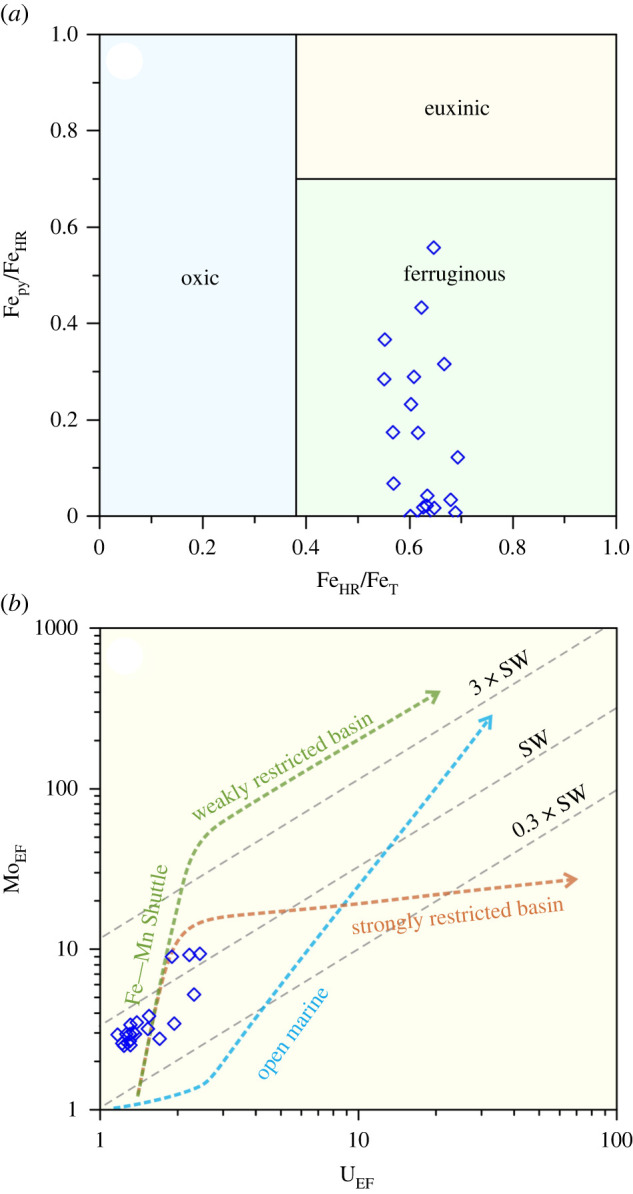
Results of the geochemical analysis on the surrounding rocks of the studied fossils. (*a*) Plots of Fe_HR_/Fe_T_ and Fe_py_/Fe_HR_ for the analysed samples; all the samples showing Fe_HR_/Fe_T_ ratios >0.38 and Fe_py_/Fe_HR_ ratios <0.7, indicating a ferruginous (or subreduced) condition. (*b*) Plots of U_EF_ and Mo_EF_ for the analysed samples; all the samples are distributed along the trend line associated with the operation of a Fe–Mn shuttle and indicate redox fluctuations between subreduced and suboxidized conditions. (Online version in colour.)

Iron speciation and trace element analyses are performed based on 20 groups of rock samples that were obtained from the same bedding planes of the studied sponge specimens. Results of the analyses are shown in figure 3 and the electronic supplementary material, table S1. The data indicate that highly reactive Fe is enriched in all the analysed samples, with Fe_HR_/Fe_T_ ratios consistently higher than 0.38. In comparison, pyrite Fe comprises only a small portion of highly reactive Fe, with Fe_py_/Fe_HR_ ratios ranging from 0 to 0.56. Trace elements Mo and U are slightly enriched compared to the upper continental crust, with EF ranging from 3 to 9 and 1 to 2, respectively.

Iron speciation results of the analysed samples are indicative of a ferruginous sedimentary environment (figure 3*a*), while the variation pattern of Mo_EF_ and U_EF_ for the samples reveals local operation of a Fe–Mn shuttle (figure 3*b*). In this case, according to the model and redox scheme proposed by Algeo & Tribovillard [45] and Algeo & Li [46], bottom waters in the study area were probably mainly ferruginous (or subreduced), while subjected to short-term redox variations to suboxidized conditions (O_2_ concentration up to 0.2 ml l^−1^; [47]).

## 4. Discussion

### (a) The unique body shape

Although the new sponge is originally preserved as pyritized spicules without soft parts (figure 1*f*; electronic supplementary material, figure S4), the complete and articulated skeletons have restored a unique body shape.

Admittedly, the body shape of sponges is across a wide range of variety [48–51]. Beside the common and idealized shapes, such as spherical, cylindrical, conical and crustose forms [49,52], there are a number of modern deep-sea sponges with peculiar morphology. For examples, glass sponges of Rossellidae and Farreidae (Hexactinellida) usually have a long, distinctive stalk and a bulged, ramulous main body part, reminiscent of a porous mushroom or a thick plume; carnivorous sponges of Poecilosclerida (Demospongiae) look like flowers of dandelions or slender tree branches with dense, tiny and horrible spinules [53]. The body shape of *Sanshapentella tentoriformis*, a stout basal trunk plus four or five conical, acute branches at the top, is obviously different from any common shape types of sponges. The characteristic tent-like branches or high peaks reveal a degree of specialization that does not match the ramulous or spiny appearances of some modern groups but stands out within coeval ancient sponges, since there are only a few of extinct sponge bodies possessing branches or other distinctive projections (such as *Hazelia* and *Vauxia* from the Burgess Shale) [19,50].

No clear oscula can be observed in the specimens of *S. tentoriformis*, possibly owing to the lateral compression. It is presumable that the exhalent opening(s) of this sponge was/were small and positioned at the top of the trunk, possibly between the branches (figure 1*b*). Spicules of *S. tentoriformis* can be classified into two morphological series (including quadripod-shaped dermal pentactines and triaxon- and stauractine-dominated substrate spicules) and three hierarchical ranks of sizes (figure 2*i*). The substrate spicules densely fill the body wall and are arranged irregularly and interlaced to each other (figures 1 and 2). This spicule distribution manner, along with the absence of prominent oscula or spongocoels, indicate that this sponge might have originally been thick-walled.

**Figure 4.**
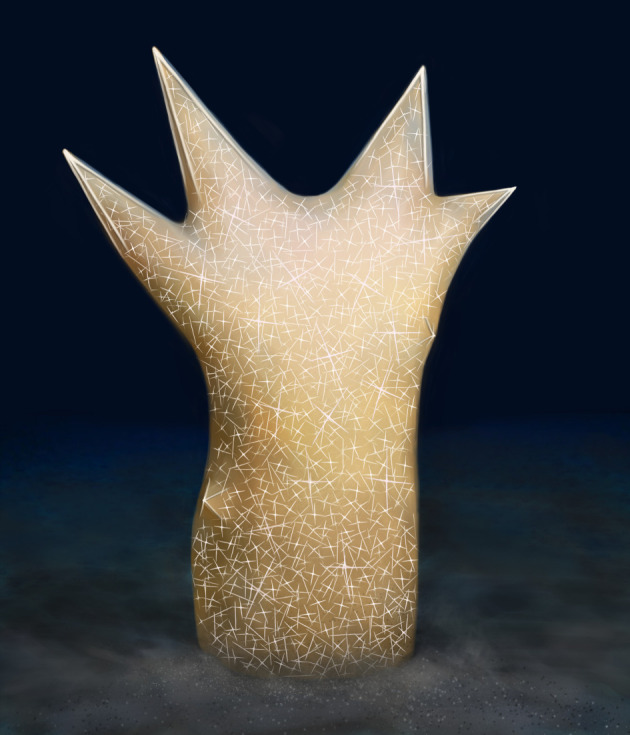
Art reconstruction of *Sanshapentella tentoriformis* sp. nov. from the Qingjiang biota. Copyright Northwest University (Online version in colour.).

Interestingly, the large dermal pentactines of *S. tentoriformis* reflect a delicate architecture that efficiently frames and fixes the body shape (figure 4). The short (degenerate), outward-pointing ray of the pentactine is positioned at the apex of the branch and four long paratangential rays form a quadripod shape with four triangular lateral faces and a low centre of gravity (figure 2*i*), which provides firmness and stability for the ostentatious conical branches. In some small specimens, robust paratangential rays of the dermal pentactines insert deeply into the body (figures 1*e* and 2*h*; electronic supplementary material, figure S2*b*,*h*), further stabilizing the branches.

### (b) Body shape diversity of the early hexactinellids

As mentioned above, the oldest complete bodies of Cambrian sponges, mostly classified into hexactinellids owing to the presence of hexactines and hexactine-derived spicules [22], are preserved in a series of black shales [12,24,25,36]. It is noted that the structure and arrangement pattern of spicules, as well as the body shape, of these sponges have already diversified, during the summit of the Cambrian Explosion (*ca* 520 Ma).

**Figure 5.**
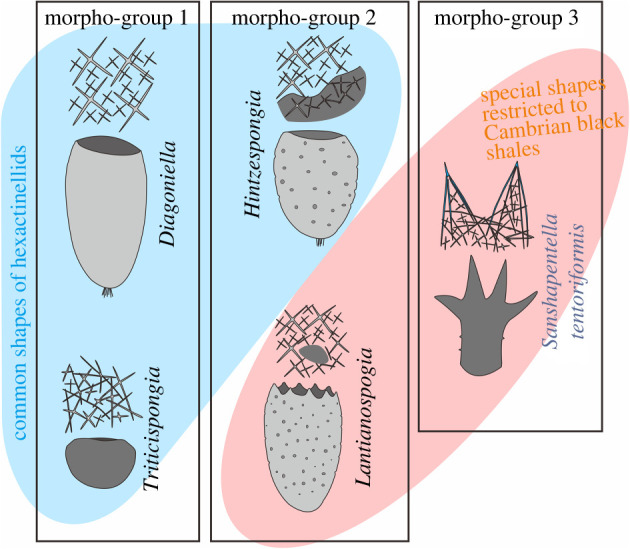
Morphological diversity and morpho-groups of representative hexactinellids from the early Cambrian black shales of South China. Black frames indicate different groups of body shapes (morpho-groups 1–3) (see the electronic supplementary material, table S3 for detail). (Online version in colour.)

Three groups of body shape (morpho-groups) are discerned within the articulated hexactinellids from the Cambrian black shale assemblages (figure 5; electronic supplementary material, table S3). Morpho-group 1 includes ovoid and cylindrical body shapes that are characterized by a relatively smooth wall and mostly a conspicuous osculum at the top. These shapes are represented by *Diagoniella*, *Protospongia* and *Triticispongia*. Morpho-group 2 refers to a barrel-like shape and an uneven body wall with numerous small, circular parietal gaps, represented by *Lantianospongia* Xiao *et al*., 2005 and *Hintzespongia*. *Lantianospongia* also has a special oscular margin with regularly spaced serrations [24]. Morpho-group 3 is revealed by *S. tentoriformis* described herein, which has a dendriform body composed of a basal trunk and a branched crown, and a body surface scattered with sharp protrusions.

Morpho-group 1 represents common body shapes of hexactinellids that adapted to various environments [13,19,22] and flourished until the present day [50,54]. The barrel-like body with circular parietal gaps (falling within morpho-group 2), such as *Hintzespongia*, also survived in other Cambrian biotas and has extant relatives [19]. While *Lantianospongia* (falling within morpho-group 2) and *S. tentoriformis* (morpho-group 3) were restricted to the Cambrian black shales of the Hetang (fragments of *S. tentoriformis* therein were described as 'undetermined form 2' [24]) and Shuijingtuo formations (electronic supplementary material, table S3).

### (c) Adaptive evolution of early sponges in response to the oxygen-deficient environment

*Sanshapentella* and other black shale sponges originally lived in a relatively deep-water, inhospitable environment where other epifauna or nektons were rare or totally absent [12,24,25,35]. The iron speciation and redox-sensitive trace element geochemistry (figure 3) indicate that the bottom water of the black shale hosting the Qingjiang biota (in the studied section) was probably dominantly ferruginous yet subjected to short-term redox fluctuations between suboxidized and ferruginous (subreduced) conditions. The presence of redox fluctuations corroborates the hypothesis that there was an 'exaerobic zone' (or 'episodically dysaerobic zone'; refers to a redox facies fluctuating between true anoxia and short periods of dysoxia) or a similar area [34,55–57] for the growth of sponges. Namely, oxygen (up to 0.2 ml l^−1^) might be episodically introduced to the subreduced-dominated bottom waters by currents, forming a localized condition with the oxygen level above the lethal threshold for sponges [31]. Furthermore, the claystone couplets (alternations of background and event beds), as the fossil horizon of the Qingjiang biota, reflect remarkable frequent storm events that caused rapid burial of the exquisite body fossils [35]. Such storms probably also facilitated oxygen-carrying currents for the deep-water sponge habitat, though the amount of oxygen was very limited and failed to oxidize the sediments or accommodate benthic animals other than sponges.

Biological experiments suggest that some modern sponges can display specific acclimation and exploit morphological, physiological and histological modifications (probably semi-permanent changes), including elongating and flattening the projecting structures (such as papillae), expanding the percentage of the aquiferous system, and reducing the cross-section area of the oscula, to cope with severe hypoxia [30,31]. These morphological changes not only contribute to increasing the surface-to-volume ratio as well as the internal water flow in order to take in more oxygen, but also reflect a trade-off between ventilation and energy cost in pumping [31]. In this view, the specialized plume-like and ramulous features of the deep-sea demosponges and hexactinellids are possibly also related to a kind of adaptive strategy coping with oxygen-deficient living conditions, though further observation and hypoxic experiments on hexactinellid sponges are required.

For Cambrian sponges, the analysis on the adaptivity relies mainly on the morphological features regarding spicule arranging and body shape building under different environmental (physical and chemical) pressures and/or competitive relationships [8,18,22,24,58]. In general, demosponges are the dominant group in the relatively shallower-water communities such as the Chengjiang, Kaili and Burgess Shale faunas, whereas hexactinellids are rare in the shallow-water environment but dominate the deep-water communities such as the Niutitang (in Hunan and Guizhou), Shuijingtuo (in Hubei) and Hetang (in Anhui) black shale sponge faunas [22]. This phenomenon is closely related to the differences in skeleton architecture between the two classes: the skeletal framework of demosponges is usually a complex network comprising bundled and interlaced spicules as well as spongin fibres, which can withstand frequent water currents; by contrast, the skeleton of hexactinellids is more regularly framed and has a lack of spongin [22,51]. Nevertheless, although not as complex as coeval demosponges in skeletal construction, the morphology of early Cambrian hexactinellids can also be classified into different groups that reflect different adaptive strategies and different levels of adaptivity in response to the environment.

Within the three morpho-groups of Cambrian hexactinellids (figure 5), morpho-group 1 adapted to a large spectrum of environments, suggesting that the ovoid and cylindrical bodies are probably one size fits all in adaptive evolution, while morpho-group 3 (represented by *S. tentoriformis*) and some extraordinary shapes of morpho-group 2 (such as *Lantianospongia*) were restricted to and dominated in the deep-water black shales, indicating that these body shapes have evolved morphological specializations in order to adapt to the inhospitable, oxygen-deficient environment. As indicated by the morphological changes of modern sponges coping with hypoxia [31] and the ramulous features of some deep-sea species of the hexactinellid family Farreidae and the demosponge order Poecilosclerida, there is a possibility that the high peaks of *S. tentoriformis* represent a dramatic elongation of projecting structures that have extended the surface area, as well as the number of ostia, of the sponge body. The parietal gaps of *Lantianospongia* and small projections of *S. tentoriformis* have also contributed to forming a rugged body surface with a relatively larger surface-to-volume ratio. From a hindsight view, these special body shapes are consequences of adaptation that helps maintain an effective use of oxygen while with a low energy cost in hypoxic conditions. By contrast, the inconspicuous oscula, and the long, ostentatious branches could hamper the water pumping rate and easily attract predators in an environment with sufficient oxygen and diverse organisms, which narrowed the habitat of *S. tentoriformis,* so that there is no similar anatomy in shallower-water communities. Therefore, the unique body shape and the tent frame-like dermal spicules, represents a stage of adaptive evolution that is specifically in response to the Cambrian black shale environment.

## Data Availability

Fossil specimens are stored in the Shaanxi Key Laboratory of Early Life and Environments (LELE), Northwest University, China. All data of the study are present in the paper and the electronic supplementary material [59].
